# Protection against *Schistosoma mansoni* infection using a *Fasciola hepatica*-derived fatty acid binding protein from different delivery systems

**DOI:** 10.1186/s13071-016-1500-y

**Published:** 2016-04-18

**Authors:** Belén Vicente, Julio López-Abán, Jose Rojas-Caraballo, Esther del Olmo, Pedro Fernández-Soto, Antonio Muro

**Affiliations:** Parasite and Molecular Immunology Laboratory, Tropical Disease Research Centre, Universidad de Salamanca (IBSAL-CIETUS), Avda. Licenciado Méndez Nieto s/n, 37007 Salamanca, Spain; Department of Pharmaceutical Chemistry, Faculty of Pharmacy, University of Salamanca, IBSAL-CIETUS, Salamanca, Spain; Present address: Centro de Investigación en Salud para el Trópico (CIST), Carretera Troncal del Caribe, Sector Mamatoco, Santa Marta, Magdalena Colombia; Present address: Facultad de Medicina, Universidad Cooperativa de Colombia, Carretera Troncal del Caribe, Sector Mamatoco, Santa Marta, Magdalena Colombia

**Keywords:** *Schistosoma mansoni*, *Fasciola hepatica*, PAL, AA0029, Fatty acid binding protein, Vaccines against helminths

## Abstract

**Background:**

Schistosomiasis is a water-borne disease afflicting over 261 million people in many areas of the developing countries with high morbidity and mortality. The control relies mainly on treatment with praziquantel. Fatty acid binding proteins (FABP) have demonstrated high levels of immune-protection against trematode infections. This study reports the immunoprotection induced by cross-reacting *Fasciola hepatica* FABP, native (nFh12) and recombinantly expressed using two different expression systems *Escherichia coli* (rFh15) and baculovirus (rFh15b) against *Schistosoma mansoni* infection.

**Methods:**

BALB/c mice were vaccinated with native nFh12 or recombinant rFh15 and rFh15 FABP from *F. hepatica* formulated in adjuvant adaptation (ADAD) system with natural or chemical synthesised immunomodulators (PAL and AA0029) and then challenged with 150 cercariae of *S. mansoni*. Parasite burden, hepatic lesions and antibody response were studied in vaccination trials. Furthermore differences between rFh15 and rFh15b immunological responses (cytokine production, splenocyte population and antibody levels) were studied.

**Results:**

Vaccination with nFh12 induced significant reductions in worm burden (83 %), eggs in tissues (82–92 %) and hepatic lesions (85 %) compared to infected controls using PAL. Vaccination with rFh15 showed lower total worm burden (56–64 %), eggs in the liver (21–61 %), eggs in the gut (30–77 %) and hepatic damage (67–69 %) using PAL and AA0029 as immunomodulators. In contrast, mice vaccinated with rFh15b showed only reductions in eggs trapped in the liver and intestine (53 and 60 %, respectively), and hepatic lesions (45 %). We observed a significant rise in TNFα, IL-6, IL-2, IL-4 and high antibody response (IgG, IgG1, IgG2a, IgM and IgE) in mice immunised with either rFh15 or rFh15b. Moreover, mice immunised with rFh15b showed an increase in IFNγ and a decrease in B220 cells compared to untreated mice, and less production of IgG1 and IgM than in mice immunised by rFh15.

**Conclusions:**

Higher level of protection is obtained by using *Fasciola hepatica*-derived FABP protein against *Schistosoma mansoni* infection. Native FABP is more effective than both recombinant systems. It could be due to post-translational modifications or FABP isoform or changes in the recombinant proteins.

**Electronic supplementary material:**

The online version of this article (doi:10.1186/s13071-016-1500-y) contains supplementary material, which is available to authorized users.

## Background

The blood flukes *Schistosoma mansoni*, *S. haematobium* and *S. japonicum* are the main causative agents of schistosomiasis in humans in Africa, Asia and South America. The Word Health Organisation (WHO) estimated that 261 million people living in 78 countries required treatment in 2013, of whom 121 million were school-aged children and 92 % lived in Africa [1]. Presently, the main strategy against schistosomiasis involves the use of praziquantel to reduce worm burden and morbidity due to its high efficacy, affordable cost, operational convenience and limited side effects [2]. However high rates of reinfection and the reduced susceptibility of schistosomula leads to sub-optimal cure rates. After decades of continuous treatment, the concern of resistant linage selection or spreading of native tolerant strains is an important threat [3]. The use of artemisinin derivatives and combinations with praziquantel could improve cure rate in endemic areas [4, 5]. Many researchers believe that immunoprophylaxis could be a promising tool together with chemotherapy, safe water supply, adequate sanitation, hygiene education or snail control [6]. Reduction of parasite burden, amelioration of pathology and blocking of transmission are considered desirable features of the vaccine [7]. The basis of vaccine use against schistosomes is demonstrated by the partial resistance developed against natural infection and the high protection induced by irradiated cercariae reaching worm reductions of 41–75 % depending on the total number of immunising parasites [8].

A plethora of proteins have been proposed as potential vaccines against schistosomiasis discovered by different methods: cDNA library screening with sera raised against whole or fractions of schistosomes, PCR amplification from a cDNA library, identification of membrane protein signal sequences, and mining the genome to identify membrane or secretory proteins by reverse vaccinology [9–11]. Only a small number of vaccines have reached Phase I clinical trials and only the glutathione-S transferase rSh28GST (Bilhvax) have reached Phase III against urinary schistosomiasis [12]. Fatty acid binding proteins (FABP) in trematodes are a family of proteins with isoforms in parenchymal and tegument cells. They are involved in cholesterol and long chain fatty acid uptake and transport, triclabendazole binding [13], anti-oxidant activity, immunomodulation [14]. Classical and non-classical such as exosomes secretory pathways were described [15]. The protein Sm14 from *S. mansoni*, derived from a cloned gene exhibited affinity to fatty acids and was able to protect outbreed mice and rabbits against the challenge with *S. mansoni* cercariae. Further research led to application of *Pichia pastoris* expression and the use of the synthetic adjuvant GLA-SE, which has been utilised in Phase I clinical trials [16]. Also, Sm14 shows a 44 % identity with rFh15 from *Fasciola hepatica* [17]. Identical basic three-dimensional structure and shared discontinuous epitopes were observed. Moreover, Sm14 induces abolition of liver damage in mice, sheep and goats against experimental infection with *F. hepatica* [16, 18, 19]. The native nFh12 and the recombinant rFh15 FABP from *F. hepatica* have shown protection in terms of reduction of worm burden and liver lesions using Freund’s adjuvant in C57/BL6 mice against *S. bovis* infection [20, 21]. Moreover, large parasite burden reduction, liver lesion amelioration and anti-fecundity effects were observed in BALB/c mice and golden hamsters vaccinated with the rFh15 using the ADAD (adjuvant adaptation) vaccination system against *S. bovis* [22, 23]. Furthermore, a FABP of 14.6 kDa purified from *Fasciola gigantica* has proved reductions in parasite counts and liver lesions against *S. mansoni* infection in CD1 mice [24]. New expression systems are needed to allow a better conservation of post-translational modifications than in prokaryotic production systems. The baculovirus-based expression system is a safe, versatile and powerful cloning tool for production of recombinant proteins in eukaryotic cells that could be interesting to test against *S. mansoni* challenge and study the immunological response [25, 26].

Immunity adjuvants are recognised to have crucial importance in vaccine development. Adjuvant adaptation (ADAD) vaccination systems was developed as an alternative to Freund’s adjuvant, which has side effects that limit its use in commercial vaccines, in vaccination against trematodes such as *F. hepatica* and schistosomes [27]. ADAD combines the antigen together with non-haemolytic saponins from *Quillaja saponaria* and a natural or synthetic immunomodulator, forming an emulsion with the non-mineral oil Montanide ISA 763AVG to obtain a long-term delivery system [27]. The natural immunomodulator PAL is a hydroalcoholic extract from the rhizome of the fern *Phlebodium pseudoaureum*, that is able to down-regulate the Th-response in mice immunised with *Anisakis simplex*, *Trichinella spiralis* and *F. hepatica* antigens [28]. The synthetic diamine AA0029 inhibits lymphoproliferation, modulates delayed-type hypersensivity in a *T. spiralis* model, modifies the ratios of CD8+, CD4+ and MHC Class II cells and increases nitric oxide production in LPS pre-stimulated rat alveolar macrophages [29]. Experiments using 14-3-3 protein from *S. bovis*, and FABP from *F. hepatica* formulated in ADAD system have yielded high protection in terms of parasite burden and liver damage [22, 23, 30].

The aim of this study is to examine the immunoprophylactic properties of three FABP from *F. hepatica* (nFh12, rFh15 and rFh15b) using the ADAD vaccination system against *S. mansoni* infection in BALB/c mice. Also immunological response to immunisation is studied using one recombinant obtained in *Escherichia coli* (rFh15) and one produced in baculovirus-transformed *Trichoplusia ni* caterpillars (rFh15b).

## Methods

### Animals, ethics statement and parasites

Animal procedures used in this study complied with the Spanish (L32/2007, L6/2013 and RD53/2013) and the European Union (Directive 2010/63/EU) regulations on animal experimentation. The Ethics Committee of the University of Salamanca (Spain) approved procedures used in the present study (protocol 48531). SPF female CD1 and BALB/c mice obtained from Charles River (Lyon, France) weighing 19–26 g used in this work were maintained in a temperature and humidity controlled environment with a 12 h light/dark cycle and provided with water and food *ad libitum* at the animal experimentation facilities of the University of Salamanca. The animals’ health status was monitored throughout the experiments by a health surveillance program according to the guidelines of the Federation of European Laboratory Animal Science Associations (FELASA). Mice were humanely euthanised with an intraperitoneal injection of pentobarbital (100 mg/kg), according to protocols supplied by the animal facilities of the University of Salamanca at the end of the experimental procedures or when any deterioration of mice health status was evidenced. Size of groups was calculated by power analysis using “size.fdr” package in R and following the 3Rs recommendations [31, 32]. All efforts were made to minimise suffering. LE strain of *S. mansoni* was maintained in our laboratory in *Biomphalaria glabrata* snails as intermediate hosts and CD1 mice as definitive hosts. The number of cercariae and their viability were determined using a stereoscopic microscope.

### *S. mansoni* soluble adult worm antigen and *F. hepatica* native nFh12 purification

Soluble adult worm antigens from *S. mansoni* (SoSmAWA) used for ELISA were prepared as previously described [20]. Twenty adult worms were suspended in 1 mL of sterile phosphate-buffered saline (PBS) containing 1 mM phenyl methyl sulphonyl fluoride (PMSF; Sigma, St Louis, MO), homogenised, frozen and thawed thrice and then sonicated thrice (70 kHz) for 1 min each. The suspension was centrifuged at 20,000 g for 30 min at 4 °C. Native 12 kDa *F. hepatica* antigen (nFh12) was purified as described by Hillyer [17] by a combination of gel filtration using Sephadex G-50 and two-step iso-electric focusing runs with 3–10 and 4–6 ampholytes. A one dimension SDS-PAGE was performed to confirm there was a single band and a rabbit monospecific, polyclonal anti-nFh12 antiserum was used in SDS-PAGE and immunoblot to confirm that the purified polypeptide was nFh12.

### Recombinant rFh15 and rFh15b protein expression and purification

The recombinant fatty-acid binding proteins from *F. hepatica* were produced using two different expression systems. The first was based on the use of *E. coli* BL21 bacteria (rFh15). The recombinant protein was manufactured following Rodríguez-Pérez et al. [33]. Briefly, total RNA from one *F. hepatica* adult worm was isolated and used for cDNA synthesis. The rFh15 gene (GenBank M95291.1) was amplified using the following primer sequences: forward 5′-GGA TCC ATG GCT GAC TTT GTG GG-3′ and reverse 5′-CTC GAG CGC TTT GAG CAG AGT G-3′ and restriction sites for *BamH*I and *Xho*I were added. PCR products were then purified and cloned into pGEX-4T2 vector with a *S. japonicum* glutathione S-transferase sequence for further detection and purification. The resulting recombinant DNA plasmid was purified and sequenced to verify the integrity of the cloned insert. Transformed *E. coli* BL21 cells were grown in Luria Bertani medium with ampicillin until reaching an optical density of 0.6 and then induced by the addition of isopropyl β-tiogalactopyranoside (IPTG) at a final concentration of 1 mM. The cell pellet was recovered by centrifugation of the culture at 18000 *g* for 30 min, suspended in PBS with 1 mM PMSF and 1 % Triton X-100 sonicated and centrifuged. Solubilised protein was purified by affinity chromatography with a glutathione Sepharose 4B resin. Non-retained proteins were eluted with PBS whilst rFh15 was eluted by addition of PBS plus thrombin. Non-retained proteins were eluted with PBS whilst the recombinant protein was cleaved to obtain rFh15 by adding 50 units of thrombin (Amersham Biosciences) in PBS. Fractions were analysed by SDS-PAGE and proteins quantified by using a Micro BCA Protein Assay Kit.

**Fig. 1:**
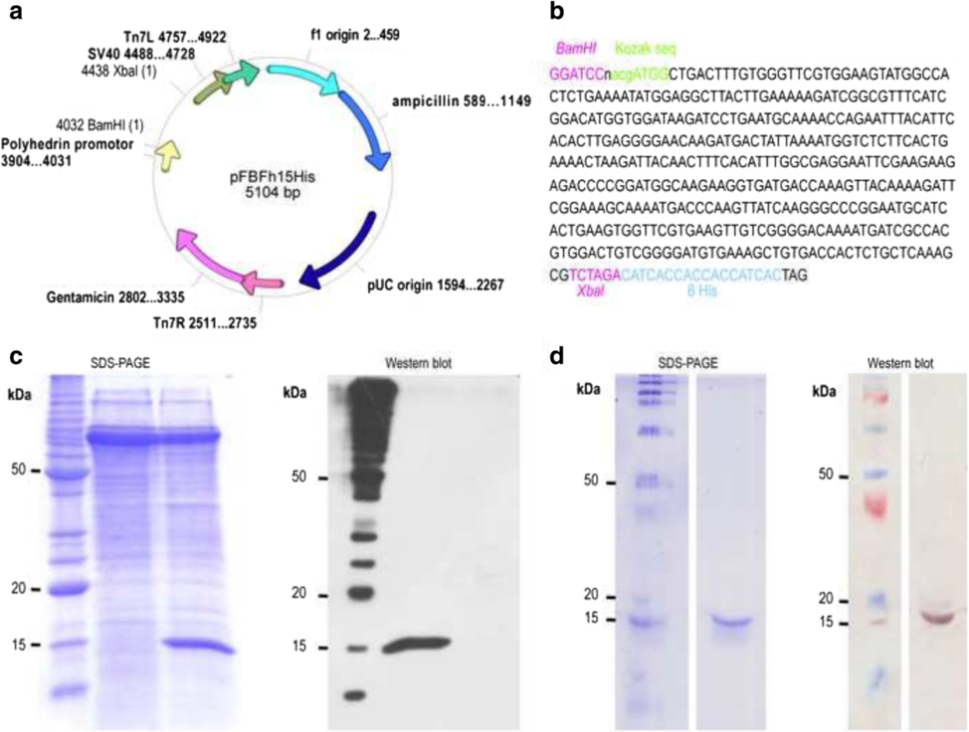
The expression and purification of rFh15b using the baculovirus system. **a** The generated vector pFBFh15His; **b** The nucleotide sequence from Fh15, including the Kozak sequence, the C-terminus 6-His tag and the restriction sequences for *BamH*I and *Xba*l; **c** The expression of rFh15b detected with Coomassie blue staining (Lane 1, molecular weight marker; Lane 2: non-induced baculovirus; Lane 3, induced baculovirus) and Western blot using anti-6His monoclonal antibody (Lane 1, molecular weight marker; Lane 2, induced baculovirus; Lane 3: non-induced baculovirus); **d** Purification of rFh15b by affinity chromatography detected by Coomassie blue staining and Western blot using anti-6His monoclonal antibody

The second method to obtain the recombinant rFh15 protein was based on the use of a baculovirus expression vector system, using standardised protocols of ALGENEX (Madrid, Spain). Briefly, to clone into pFasBacHis vector a nucleotide sequence from 15 kDa FABP protein (GenBank M95291.1) was synthesised and a Kozak sequence was inserted into the N-terminus extreme, along with *BamH*I and *Xba*I restriction sites at the N- and C-terminus respectively. The plasmid pMA (ampR) with the cloned Fh15 gene between KpnI/Sacl sites was used to amplify DNA by transformation of *E. coli* (DH5alpha) cells and isolation of ampicillin-resistant colonies. The resulting amplified DNA together with the cloning vector (pFasBacHis) were cut with restriction enzymes *BamH*I and *Xba*l and the corresponding band (412 bp) from Fh15 insert was isolated and purified. pFasBacHis vector was dephosphorylated with alkaline phosphatase treatment and the Fh15 insert was subsequently ligated. The resulting product was then used to transform *E. coli* (DH5alpha) cells and ampicillin- and gentamicin-resistant colonies were then isolated. The DNA from these isolated colonies was isolated and characterised using the restriction enzymes for *BamH*I and *Xba*l sites, respectively, and automated sequence was performed to verify the sequence of the insert. The resulting vector and the sequence of the Fh15 insert is depicted in Fig. 1a. To obtain the recombinant baculovirus, *E. coli* special competent (DH10B) cells were transformed starting from a previously generated vector (pFBFh15His). These cells carry the receptor bMON14272 that contains a beta-galactosidase codifying gene. Upon incorporation in the same cell vector and receptor, the recombinant baculovirus presents resistance to kanamycin, tetracyclin and geneticin and loses its beta-galactosidase activity. One colony resistant to the three antibiotics was selected and the DNA was isolated and used to transfect insect cells sf21 using the cellfectin reagent (Invitrogen, Waltham, MA USA). Seventy-two hours after the transfection, the so-called progeny 1 from the recombinant baculovirus was collected and stored until further use. Finally, thirty *Trichoplusia ni* larvae were inoculated with the previously obtained recombinant virus. Larvae were harvested during the next 48–96 h and the expression of the recombinant protein was assessed using both Coomassie blue staining and Western blot with monoclonal anti-6His antibodies.

### ADAD vaccination system

The rFh15 protein was formulated in a micelle composed of non-haemolytic saponins from *Quillaja saponaria* (Qs; Sigma, St Louis, Missouri, USA) and natural (PAL) or synthetic aliphatic diamine (AA0029) as immunomodulator. Thereafter, this micelle was emulsified in a non-mineral oil (Montanide ISA763A, SEPPIC, Paris, France) at an oil/water ratio of 70/30 and subcutaneously injected into BALB/c mice. The ADAD vaccination system consists of a set of two subcutaneous injections. The first injection, called “Adaptation”, contains Qs and PAL or AA0029 emulsified in the non-mineral oil. The second injection, administered 5 days after the adaptation, contains the rFh15 antigen with Qs and PAL or AA0029 in the emulsion oil. Individual doses per injection included in each case were as follows: 20 μg of Qs, 600 μg of PAL or 100 μg of AA0029, and 10 μg of nFh12, rFh15 or rFh15b, resulting in a final volume of a 200 μl injection of emulsion in the non-mineral oil [22, 30].

### Vaccination experiment schedules

BALB/c mice were randomly allocated in groups of nine animals each as follows: Untreated and uninfected; *S. mansoni* infected; Control adjuvant (injected with ADAD with Qs and the natural immunomodulator PAL or the synthetic AA0029) and Vaccinated groups (vaccinated with ADAD with the corresponding FABP nFh12, rFh15 or rFh15b formulated with the corresponding immunomodulator PAL or AA0029 and infected). Two weeks after the first immunisation animals were boosted with the same doses. Two weeks after the second immunisation, each mouse was exposed to 150 cercariae of *S. mansoni* for 45 min. Eight weeks post-infection all mice were euthanised with intraperitoneal injection of sodium pentobarbital (100 mg/kg) and then perfused by intra-cardiac injection of PBS plus heparin, and the number of *S. mansoni* adult worms recovered from the portal and mesenteric veins was recorded. In addition, the number of parasite eggs in the liver and intestine was counted using a McMaster camera after digestion with 25 ml of 5 % KOH for 16 h at 37 °C with gentle shaking. Macroscopic lesions of the liver were quantified as granuloma-affected surface per 100 mm^2^ in each mouse using ImageJ 1.45 s software [34]. Protection percentage was calculated for all parasitological and pathological magnitudes as follows: (mean in the infected control group – mean in experimental group) × 100/mean in infected control group. Blood samples were collected from each animal before immunisation, infection and necropsy for humoral immune response studies.

### Specific antibody response against FABP and SoSmAWA

Specific anti-rFh15 or anti-SoSmAWA antibody profiles were measured using an indirect ELISA as described by Abán et al. [20]. Briefly, 96-well polystyrene plates (Costar) were coated with 2.0 μg of nFh12, rFh15, rFh15b or 2.5 μg of SoSmAWA antigen for 12 h in carbonate buffer (pH 9.0) and then blocked with 2 % bovine serum albumin in PBS. Sera were then added at 1:100 dilutions and incubated for 1 h at 37 °C, followed by the addition of goat peroxidase-labelled anti-mouse IgG, IgG1, IgG2a, IgM or IgE antibodies at 1:1000 dilution (Sigma, St. Louis, MO, USA). The reaction was developed with H_2_O_2_ and ortophenilenediamine (OPD, Sigma) in citrate buffer (pH 5.0) and absorbance was measured at 492 nm with an Ear400FT ELISA reader (Lab Instruments, Groding, Austria).

### Immune response in BALB/c mice immunised with the recombinant FABP rFh15 and rFh15b

Four groups of six female BALB/c each were used for the characterisation of immunological response: Untreated; Injected with ADAD only with AA0029+Qs as adjuvant control; Immunised with rFh15 formulated in ADAD system with AA0029 (AA0029+Qs+rFh15); and Immunised with rFh15b formulated in ADAD system with AA0029 (AA0029+Qs+rFh15b). Mice were immunised and two booster doses were given after 2 and 4 weeks, respectively. Two weeks after the immunisation schedule all mice were anesthetised with isoflurane and euthanised by cervical dislocation. Spleens were then aseptically removed for obtaining splenocytes by perfusion with sterile PBS to study cytokine profile and to quantify T-cell subpopulations. Blood samples were collected for antibody detection from the animals before each immunisation and at the necropsy.

### Cytokine measurement

Splenocytes obtained from individual mice were cultured in a 6-well plate at 1 × 10^6^ cells per well in a complete RPMI 1640 medium containing 10 % heat-inactivated foetal calf serum, 5 mM L-glutamine and antibiotics: 100 units/ml penicillin and 100 μg/ml streptomycin as previously described [35]. Cells were *in vitro* stimulated with rFh15 or rFh15b at a final concentration of 10 μg/ml for 72 h at 37 °C in a humidified atmosphere with 5 % CO_2_. Culture supernatants were recovered for cytokines determination. Splenocytes belonging to untreated mice were used as controls. A flow cytometry-based technique was used for interferon γ (IFNγ), tumor necrosis factor α (TNFα), interleukin (IL) 1α, IL-2, IL-4, IL-6, IL-10 and IL-17 quantitation in each of the groups of mice used in this study. The FlowCytomix Mouse Th1/Th2 10plex kit (Bender MedSystems GmbH, Vienna, Austria) was used according to the manufacturer’s instructions. Briefly, different size fluorescent beads, coated with capture antibodies specific for the aforementioned cytokines, were incubated with mouse splenocyte samples and with biotin-conjugated secondary antibodies for 2 h at room temperature. The specific antibodies bound to the analytes captured by the first antibodies. After washing the tubes with PBS plus 2 % foetal calf serum, Streptavidin-Phycoerythrine (S-PE) solution was added and incubated at room temperature for 1 h. S-PE binds to the biotin conjugate and emits fluorescent signals. Flow cytometry data was collected using a FACSCalibur flow cytometer (BD Biosciences, Franklin Lakes, NJ, USA) at the University of Salamanca’s Flow Cytometry Central Service; 8000 events were collected (gated by forward and side scatter) and data was analysed using FlowCytomix Pro 3.0 software (Bender MedSystems, Vienna, Austria). Each cytokine concentration was determined from standard curves using known mouse recombinant cytokine concentrations.

### Flow cytometry analysis of splenic B and T-cell populations

Splenocytes from untreated, AA0029+Qs-treated, rFh15-immunised and rFh15b-immunised mice were incubated with the blocking anti-CD16/CD32 monoclonal antibody for 5 min at room temperature and stained with commercial fluorochrome-conjugated antibodies at 1/50 dilution in PBS plus 2 % foetal calf serum for 30 min at 4 °C. Rat anti-mouse CD45-peridinin chlorophyll protein (PerCP)- cyanine dye (Cy5.5), CD4-fluorescein isothyosanate (FITC), CD8-phycoerythrin (PE), CD45R/B220-allophycocyanin (APC), CD197-PE (CCR7), CD62L-APC and hamster anti-mouse CD27 APC (BD Pharmingen, USA) were used. After incubation, cells were washed in PBS with 2 % foetal calf serum and centrifuged at 1000 *g* for 5 min and the supernatant was then discarded. The cells were fixed with 100 μl of a 2 % paraformaldehyde solution for 1 h at 4 °C. Phenotypic analyses were performed in a FACScalibur flow cytometer. Data were collected on 30,000 events (gated by forward and side scatter) and analysed using Gatelogic Flow Cytometry Analysis Software (INIVAI technologies Pty Ltd).

### Statistical analysis

The results were expressed as the mean ± standard error of the mean (SEM). Normal distribution of data was assessed by Kolmogorov-Smirnov test and homogeneity of variance was tested by Barrett test. Significant differences among groups were found using one-way ANOVA test and *post-hoc* Tukey’s honest significance tests (HSD) or Kruskal-Wallis (K-W) test. All statistical analyses were considered significant at *P* < 0.05. SPSS 21 software (IBM) was used for data analysis.

## Results

### Recombinant expression and detection of antigens

Expression and purification of *F. hepatica*-derived native nFh12 and recombinant rFh15 proteins were previously reported [17]. Here, we used a baculovirus expression vector system that improves the production of recombinant proteins compared to the classical expression systems based on the use of bacteria or yeasts, which also retains recombinant proteins native configuration along the production and purification steps to produce a *F. hepatica*-derived FABP protein (Fig. 1a and 1b). Starting with 30 *T. ni* larvae inoculated with recombinant virus, cells were recovered during the next 48–96 h to assess recombinant protein expression, which was confirmed using both Coomassie blue staining and western blot using anti-6His monoclonal antibody as shown in Fig. 1c. Coomassie blue staining detected a majority band with an estimated molecular weight of 15.7 kDa in the crude extract. Specific detection with monoclonal antibody confirmed the presence of a single band with the same molecular weight. Upon detection, the recombinant protein was on-column purified by affinity chromatography using a Ni-NTA column (Fig. 1d). As depicted, a single band with a molecular weight of 15 kDa was detected using Coomassie blue staining, coming from pooled column-retained fractions, dialysed against ammonium carbonate (50 mM), lyophilised and resuspended in high-purity distilled water. Western blot from the same fraction also revealed the presence of a single band with the same molecular weight (Fig. 1d). Protein quantitation revealed the recovery of 5 mg of pure recombinant protein.

### Vaccination with the native nFh12 formulated in ADAD with PAL triggers protection against *S. mansoni* infection

**Fig. 2:**
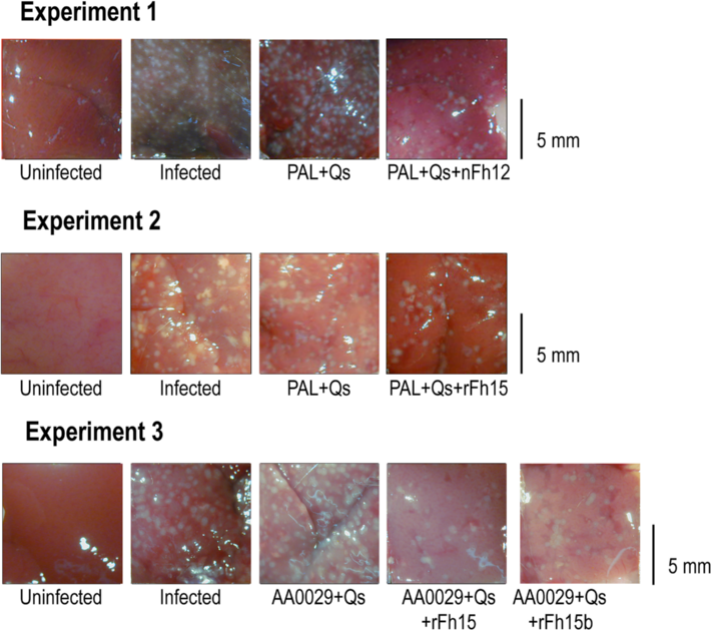
Representative hepatic lesion area reduction in BALB/c mice after vaccination. Natural and recombinant FABP (nFh12, rFh15 or rFh15b) formulated with the adjuvant adaptation (ADAD) vaccination system were used with the natural immunomodulator PAL or the synthetic AA0029 and challenged with 150 cercariae of *S. mansoni* in three separate experiments

**Fig. 3:**
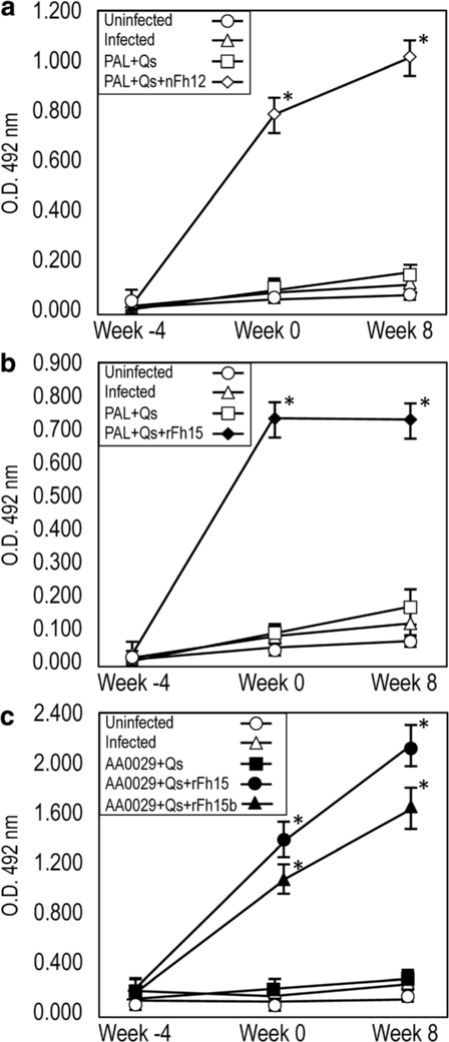
Serum-specific IgG antibody levels by ELISA during vaccination trials against nFh12, rFh15 or rFh15b. Data presented as the mean ± standard error of the mean. BALB/c mice were vaccinated with their respective antigens formulated with the adjuvant adaptation (ADAD) vaccination system with the natural immunomodulator PAL or the synthetic AA0029 and challenged with 150 cercariae of *S. mansoni*. **a** Vaccination using nFh12 formulated with PAL; **b** Vaccination with rFh15 using PAL; **c** Vaccination using rFh15 or rFh15b formulated with AA0029. O.D., optical densities; *P < 0.05 compared to uninfected controls

**Fig. 4:**
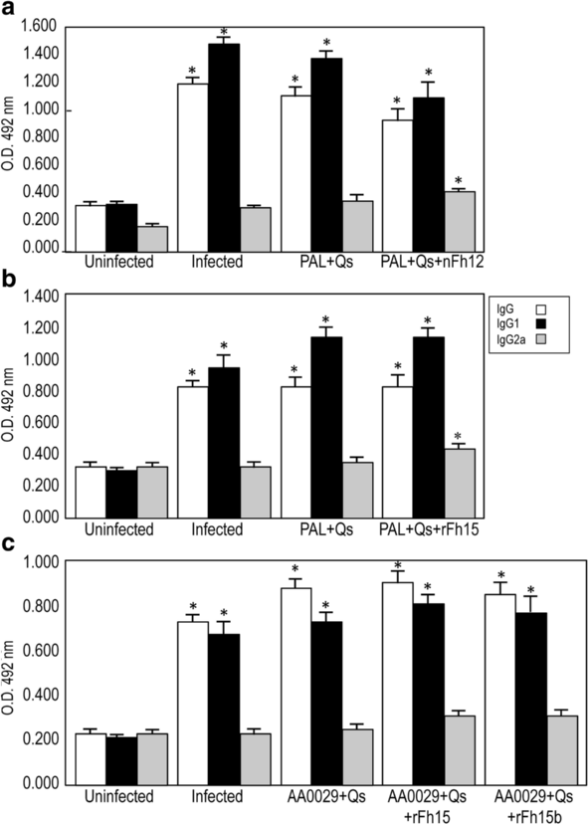
Serum-specific IgG, IgG1 and IgG2a antibody levels by ELISA 8 weeks post-challenge against soluble adult worm antigens from *S. mansoni* (SoSbAWA). Data presented as the mean ± standard error of the mean. BALB/c mice were vaccinated with their respective antigens formulated with the adjuvant adaptation (ADAD) vaccination system with the natural immunomodulator PAL or the synthetic AA0029 and challenged with 150 cercariae of *S. mansoni*. **a** Vaccination with PAL+Qs+nFh12+PAL; **b** Vaccination with PAL+Qs+rFh15; **c** vaccination using AA0029+Qs+rFh15 and AA0029+Qs+rFh15b. O.D., optical densities; *P < 0.05 compared to uninfected controls

Significant reductions in recovered total worms (83 %), males (87 %) and females (82 %) were observed in BALB/c mice immunised with nFh12 formulated in ADAD with the natural immunomodulator PAL (PAL+Qs+nFh12) compared to the infected control group (Table 1). Also, a significant decrease in the number of eggs present in the liver (82 %) and in the intestine (92 %) were detected (Table 1). In concordance, hepatic damage extension was significantly reduced (85 %) compared to the infection control group (Table 1, Fig. 2). Furthermore, mice injected only with PAL+Qs did not show significant protection in terms of parasite burden or hepatic lesions (Table 1). A significantly higher production of specific anti-nFh12 IgG was observed in the nFh12 vaccinated group compared to uninfected, infected or adjuvant controls after the second immunisation which remained until the end of the experiment (Fig. 3a and Additional file 1: Table S1). Also all infected groups showed a significant production of IgG, IgG1 8 weeks post-infection against SoSmAWA but only vaccinated with PAL+Qs+nFh12 showed significant IgG2a production (Fig. 4a and Additional file 2: Table S2).

**Table 1:** Protection levels (% of reduction, R) in worm recovery (total counts, females and males), hepatic damage extension (mm^2^/100 mm^2^) and number of eggs per gram (EPG) in the tissues in vaccinated BALB/c mice using natural and recombinant FABP (nFh12, rFh15 or rFh15b) formulated with the adjuvant adaptation (ADAD) vaccination system with the natural immunomodulator PAL or the synthetic AA0029. Data presented as the mean ± standard error of the mean. ANOVA *F-* and *P-*values, and *post-hoc* Tukey’s honest significance test *P* values of significant increases are included

Groups	Total worms	R	Females	R	Males	R	Hepatic lesion	R	EPG in liver	R	EPG intestine	R
	(mean ± SEM)	(%)	(mean ± SEM)	(%)	(mean ± SEM)	(%)	(mean ± SEM)	(%)	(mean ± SEM)	(%)	(mean ± SEM)	(%)
Experiment 1												
Infected	36.3 ± 4.9	–	19.7 ± 2.9	–	16.6 ± 2.0	–	64.1 ± 7.1	–	17,432 ± 3,586	–	14,812 ± 3,934	–
PAL+Qs	23.4 ± 2.5	36	12.4 ± 1.4	37	11.1 ± 1.0	33	74.4 ± 6.4	NR	16,551 ± 2,620	5	17,367 ± 2,277	NR
PAL+Qs+nFh12	6.0 ± 1.7 *P* < 0.001^a^	83	3.6 ± 1.0 *P* < 0.001^a^	82	2.1 ± 0.8 *P* < 0.001^a^	87	9.4 ± 3.0 *P* < 0.001^a^	85	3,089 ± 1,001 *P* = 0.001^a^	82	1,186 ± 523 *P* < 0.001^a^	92
ANOVA	*F*_(2,24)_ = 23.39 *P* < 0.001		*F*_(2,24)_ = 18.49 *P* < 0.001		*F*_(2,24)_ = 30.13 *P* < 0.001		*F*_(2,24)_ = 39.41 *P* < 0.001		*F*_(2,24)_ = 10.27 *P* < 0.001		*F*_(3,32)_ = 16.31 *P* < 0.001	
Experiment 2												
Infected	49.0 ± 6.1	–	24.0 ± 7.3	–	25.0 ± 8.8	–	61.2 ± 8.3	–	18,008 ± 2,362	-	18,197 ± 2,079	–
PAL+Qs	31.6 ± 3.4	35	18.6 ± 2.1	22	13.5 ± 1.2	46	71.0 ± 6.2	NR	17,098 ± 2,706	5	21,700 ± 2,968	NR
PAL+Qs+rFh15	21.8 ± 2.5 *P* = 0.006^a^	56	9.0 ± 1.2 *P* < 0.001^a^	63	12.8 ± 1.5 *P* < 0.001^a^	49	18.7 ± 2.2 *P* < 0.001^a^	69	14,247 ± 668	21	12,724 ± 488	30
ANOVA	*F*_(2,24)_ = 9.04 *P* < 0.001		*F*_(2,24)_ = 13.80 *P* < 0.001		*F*_(2,24)_ = 9.93 *P* < 0.001		*F*_(2,24)_ = 18.37 *P* < 0.001					
Experiment 3												
Infected	34.5 ± 6.9	–	18.0 ± 3.6	–	16.5 ± 3.4	–	61.8 ± 14.4	–	9,986 ± 2,360	–	7,748 ± 1,315	–
AA0029+Qs	42.5 ± 8.0	NR	20.0 ± 4.4	NR	22.5 ± 3.9	NR	77.0 ± 19.0	NR	13,242 ± 1,597	NR	8,084 ± 775	NR
AA0029+Qs+rFh15	12.5 ± 3.8 *P* = 0.046^a^	64	5.5 ± 2.0 *P* = 0.011^a^	69	7.0 ± 2.0 *P* = 0.041^a^	58	20.6 ± 14.0 *P* = 0.049^a^	67	3,872 ± 1,814 *P* = 0.024^a^	61	1,800 ± 730 *P* = 0.001^a^	77
AA0029+Qs+rFh15b	25.1 ± 7.8	27	10.1 ± 3.2 *P* = 0.021^a^	44	15.0 ± 4.8	9	15.2 ± 7.1 *P* = 0.048^a^	75	4,692 ± 1,181 *P* = 0.035^a^	53	3,098 ± 800 *P* = 0.001^a^	60
ANOVA	*F*_(3,32)_ = 3.09 *P* = 0.047		*F*_(3,32)_ = 3.75 *P* = 0.026		*F*_(3,32)_ = 2.97 *P* = 0.048		*F*_(3,32)_ = 4.05 *P* = 0.007		*F*_(3,32)_ = 4.25 *P* = 0.003		*F*_(3,32)_ = 11.61 *P* < 0.001	

*NR* no-reduction: ^a^Significant differences in comparison with infected controls

### Vaccination with the recombinant rFh15 formulated with ADAD using PAL stimulates high protection against *S. mansoni* infection

Mice vaccinated with rFh15 formulated in ADAD with the natural immunomodulator PAL (PAL+Qs+rFh15) induced significant reduction in worm burden (56 % in total worms, 63 % in females and 49 % in males) compared to infected controls (Table 1). Slight decreases in the number of eggs present in the liver (21 %) and the gut (30 %) of the vaccinated group were observed in comparison to infected group (Table 1). Moreover, in concordance with the reduction in worm burden, liver surface damage showed significant reduction (69 %) compared to infected group (Table 1, Fig. 2b). Mice injected with PAL+Qs did not show significant reductions in parasite burden or hepatic lesions (Table 1). A significantly higher production of specific anti-rFh15 IgG, was observed in the rFh15 vaccinated group compared to the uninfected control group (Fig. 3b and Additional file 1: Table S1). Also all infected groups showed significant increase of IgG, IgG1 against SoSmAWA 8 weeks post-infection, but only groups vaccinated with PAL+Qs+Fh15 showed statisticaly significant IgG2a increase (Fig. 4b and Additional file 2: Table S2).

### Vaccination with rFh15 induces more protection than rFh15b against *S. mansoni* infection in BALB/c mice using ADAD vaccination system with the immunomodulator AA0029

Vaccination with rFh15 formulated in ADAD with the synthetic immunomodulator AA0029 (AA0029+Qs+rFh15) induces significant reduction of worm burden (64 % in total worms, 69 % in females and 58 % in males) in comparison with infected controls (Table 1). Also significant decreases in the number of eggs recovered from the liver (61 %) and gut (77 %) of the vaccinated group were observed in comparison with the infected group agreeing with the reduction in worm burden (Table 1). Moreover, there was a significant reduction (67 %) of liver surface damage in vaccinated mice compared to infected mice (Table 1, Fig. 2). Vaccination with rFh15b obtained from *T. ni* larvae (AA0029+Qs+rFh15b) showed significant protection in terms of recovered females (44 %), eggs confined in liver (53 %), eggs in the gut (60 %) and hepatic lesions (75 %) However, no significant reduction was observed in the recovery of total and male adult parasites (Table 1, Fig. 2). Adjuvant controls treated with AA0029+Qs showed no protection against *S. mansoni* challenge (Table 1). A significantly higher production of specific anti-rFh15 and anti-rFh15b IgG was observed in the vaccinated group compared to the uninfected control group at the time of infection and at the end of the experiment particularly in mice vaccinated with rFh15 (Fig. 3c and Additional file 1: Table S1). Also all infected groups showed significant increase in of IgG, IgG1 against SoSmAWA 8 weeks post-infection but not IgG2a (Fig. 4c and Additional file 2: Table S2).

### Cell immune response induced by rFh15 and rFh15b using ADAD vaccination system with AA0029 as an immunomodulator

Cytokine levels were measured in cultured splenocyte supernatants to analyse Th1, Th2, Treg and Th17 T-cell responses. It was observed that mice immunised with AA0029+Qs+rFh15 showed a significant increase in TNFα, IL-6, IL-2 and IL-4 compared to untreated and adjuvant controls (Table 2). Similarly, mice treated with AA0029+Qs+rFh15b had high levels of TNFα, IL-2 and IL-4 compared to untreated and adjuvant controls (Table 2). Additionally, we observed less IL-6 production and highly significant levels of IFNγ than mice vaccinated with AA0029+Qs+rFh15 (see Table 2). We observed that untreated mice and adjuvant controls (PAL+Qs) showed similar cytokine patterns. Also, no differences were found in IL-17 and IL-10 cytokine levels in either rFh15- or rFh15b-immunised mice. Regarding the percentage of splenocyte populations, only mice vaccinated with AA0029+Qs+rFh15b showed a significant reduction in B220 cells compared with untreated and PAL+Qs treated animals (Table 3). No differences in T and B splenocyte population were observed between untreated mice and those treated with PAL+Qs.

**Table 2:** Cytokine production (TNF-α, IL-6, IL-1α IFNγ, IL-2, IL-4, IL-10, IL-17) in supernatants of splenocyte cultures in untreated BALB/c mice, treated with AA0029+Qs and immunised with AA0029+Qs+rFh15 and AA0029+Qs+rFh15b 2 weeks after immunisation schedule. Data are presented as the mean ± standard error of the mean. Kruskal-Wallis *χ* ^*2*^, degrees of freedom (*df*) and *P-*values, and *P-*values in case of significant differences in pairwise comparisons

Cytokine	Untreated	AA0029+Qs	AA0029+Qs+rFh15	AA0029+Qs+rFh15b	Kruskal-Wallis		
	(pg/ml)	(pg/ml)	(pg/ml)	(pg/ml)	*χ* ^*2*^	*df*	*P*
TNFα	313 ± 98	214 ± 20	937 ± 130	1,074 ± 89	16.22	3	0.001
			*P* = 0.001^a^	*P* = 0.001^a^			
IL-6	964 ± 118	1,318 ± 137	2,755 ± 226	1,613 ± 137	17.11	3	0.001
			*P* = 0.001^a^	*P* = 0.001^a^			
				*P* < 0.001^b^			
IL-1α	527 ± 65	368 ± 32	448 ± 23	581 ± 142			
IFNγ	543 ± 35	643 ± 16	735 ± 23	890 ± 79	20.40	3	< 0.001
				*P* < 0.001^a^			
				*P* < 0.001^b^			
IL-2	592 ± 74	774 ± 84	1,025 ± 47	888 ± 41	10.27	3	0.016
			*P* < 0.001^a^	*P* = 0.001^a^			
IL-4	1,138 ± 101	1,508 ± 82	2,078 ± 145	1,653 ± 18	16.91	3	<0.001
			*P* < 0.001^a^	*P* < 0.001^a^			
IL-10	481 ± 46	485 ± 39	424 ± 7	459 ± 21			
IL-17	1,724 ± 167	2,048 ± 43	2,053 ± 46	1,988 ± 268			

^a^Significant differences in comparison with untreated group; ^b^Significant differences compared to AA0029+Qs+rFh15 vaccinated group

**Table 3:** Percentages of splenocyte populations (CD45, CD4, CD8, CD197, CD62L, CD27, B220) in untreated BALB/c mice, treated with AA0029+Qs and immunised with AA0029+Qs+rFh15 and AA0029+Qs+rFh15b 2 weeks after immunisation schedule. Data presented as the mean ± standard error of the mean

Cell population	Untreated (%)	AA0029+Qs (%)	AA0029+Qs+rFh15 (%)	AA0029+Qs+rFh15b (%)
CD45	75.7 ± 3.4	77.0 ± 0.7	75.5 ± 2.8	66.7 ± 1.3
CD4	21.1 ± 1.3	20.7 ± 0.4	21.3 ± 0.5	21.7 ± 4.0
CD8	8.4 ± 0.5	8.4 ± 0.6	10.2 ± 0.6	9.5 ± 1.3
CD197	16.9 ± 1.7	18.0 ± 2.1	12.6 ± 2.8	14.9 ± 0.6
CD62L	23.2 ± 3.2	20.1 ± 5.0	17.2 ± 0.9	15.2 ± 5.0
CD27	19.4 ± 1.9	18.0 ± 1.6	16.9 ± 0.8	16.7 ± 3.6
B220	35.9 ± 3.2	39.4 ± 0.6	23.2 ± 1.7	21.3 ± 0.7*

*Significant differences in comparison with untreated group *P* < 0.001 (Kruskal-Wallis *χ* ^*2*^ = 15.51, *df* = 3, *P* = 0.001)

### Differential antibody patterns in mice vaccinated with rFh15 *vs* rFh15b

**Fig. 5:**
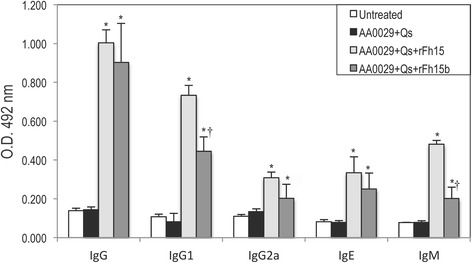
Antibody detection (IgG, IgG1, IgG2a, IgE and IgM) against rFh15 or rFh15b 2 weeks after immunisation schedule in BALB/c mice. Data presented as the mean ± standard error of the mean. Groups: Untreated; Treated with AA0029+Qs; Immunised with AA0029 + Qs + rFh15; and Immunised with AA0029+Qs+rFh15b. O.D., optical density; *P < 0.05 in comparison with untreated controls and treated with AA0029+Qs; ^†^ *P* < 0.05 compared to mice treated with AA0029+Qs+rFh15

Antibody response of rFh15- and rFh15b-immunised mice were studied to understand the intensity of the humoral response elicited by the two recombinant proteins, due to the importance of antibodies in resistance to schistosomiasis and in an attempt to explain the differential protection observed between these molecules. Two weeks after the immunisation schedule, a significantly higher production of specific IgG, IgG1, IgG2a, IgM, IgE anti-rFh15 or anti-rFh15b was observed in AA0029+Qs+rFh15 and in AA0029+Qs+rFh15b vaccinated mice respectively, compared to untreated controls (Fig. 5 and Additional file 3: Table S3). Furthermore, we observed significantly higher levels of IgG1 and IgM in mice vaccinated with rFh15 than mice vaccinated with rFh15b (Fig. 5 and Additional file 3: Table S3).

## Discussion

A significant effort has been focused on schistosomiasis vaccine development because of its potential ability to control or eradicate the disease. FABP from *F. hepatica* have demonstrated a valuable cross-protection against *S. bovis* in experimental models [20–23]; similar effects were reported for FABP of 14.6 kDa from *F. gigantica* [24]. Also, the *S. mansoni* FABP Sm14 have reached Phase I studies [19]. However, the immunoprotective potential of *F. hepatica* FABPs has not been tested against *S. mansoni* infection until now. In this study, we present the immunoprotective potential of FABP obtained from *F. hepatica* represented by the native form (nFh12) and two recombinants (rFh15, rFh15b) against the *S. mansoni* infection in BALB/c mice. These molecules have been expressed in prokaryotic and eukaryotic systems. The inbred mice have a biased Th2 genetic background, resembling the immunological profile observed in people living in endemic areas [36]. In this study, we used the adjuvant adaptation (ADAD) vaccination system using natural (PAL) and synthetic (AA0029) immunomodulators developed by our research group for vaccination against fasciolosis and schistosomiasis to improve limitations of the classical Freund’s adjuvant [27, 30, 35, 37].

We observed a high protection in terms of worm recovery, eggs trapped in the tissues and hepatic damage in mice vaccinated with the native nFh12 and the *E. coli* recombinant rFh15. These results are close to those obtained by vaccination against *S. bovis* with both antigens formulated in ADAD vaccination system with PAL as well as AA0029 [22, 23]. Our results are also comparable to those shown using the FABP Sm14 obtained from *S. mansoni* in experimental models [16] or using the *F. gigantica* 14.6 kDa molecule [24]. Taken together, these results reinforce the value of FABPs in schistosomiasis vaccination development. We observed a high production of specific IgG by ELISA against the three antigens used for vaccination, indicating an intense immunological response. A vigorous humoral response is found in natural resistance to infection possessed by people living in hyperendemic areas [36, 38, 39] and experimental models [40]. Also, vaccinated animals generated high levels in both IgG and IgG1 against SoSmAWA 8 weeks post-challenge, but there was significant production of IgG2a only using the natural immunomodulator PAL. This effect has been observed in previous studies related to the use of PAL in vaccination against *F. hepatica* and *S. bovis* and its association with protection and downregulation of the dominant Th2 established in schistosomes or *F. hepatica* infections [22, 23, 41, 42]. An appropriate adjuvant system able to induce an adequate immune response is recognised as an important tool for developing vaccines. A good feature is the specific adjuvant activity driving the immunological response together with the antigen [36, 43]. We did not find any protection induced in mice treated either with PAL+Qs or AA0029+Qs after challenge with *S. mansoni* when compared with infection controls. This indicates the specific activity of both adjuvants in our experiments.

Additionally, we observed that the antigen obtained using baculovirus as vector (rFh15b) formulated with AA0029 in ADAD showed high reductions of eggs in the tissues and liver damage, but there were only small, non-significant reductions in total worm burden compared to AA0029+Qs+rFh15-vaccinated mice after the challenge. Then we studied the immune response induced with rFh15 and rFh15b using the synthetic immunomodulator AA0029 along *E. coli* recombinant rFh15 in the ADAD vaccination system. We observed that it promoted an early potent mixed Th1/Th2 pro-inflammatory immune response with significant production of TNFα, IL-6, IL-2, IL-4 and high level of specific antibodies. All of this could explain the protection against *S. mansoni* challenge as pointed out in previous studies of experimental protection against *F. hepatica* and *S. bovis* using AA0029 formulated in ADAD [23, 35, 36]. Vaccination with the protein produced in *T. ni* (AA0029+Qs+rFh15b) showed high levels of TNFα, IL-6, IFNγ IL-2, IL-4 and antibodies, but there was a reduction in B220 cells percentage compared to untreated mice. Moreover, we observed less IL-6, IgG1 and IgM in these mice compared to those immunised with AA0029+Qs+rFh15. This indicates a potent pro-inflammatory and Th1/Th2 mixed response with an impairment of humoral response involving B memory cells and immunoglobulins that could be responsible for the low protection in terms of worm recovery [44]. Another possible explanation of the differences in protection could be the post-translational modifications that occur in the different expression systems involving glycosylation and the fact that only one isoform was used [45].

## Conclusions

In conclusion, our data demonstrated the ability of FAPB obtained from *F. hepatica* to induce protection against infections with *S. mansoni* in BALB/c mice. Also the use of PAL seems to induce an increase in Th1-like immune response during infection. ADAD formulation with the immunomodulator AA0029 showed an intense pro-inflamatory and mixed Th1/Th2 immune response. These molecules may have valuable effects leading to reduction of pathology and transmission of the disease. Our results warrant further studies in other animal models closer to humans to state the actual protection ability of FABP against *S. mansoni* infection.

## Additional files

Additional file 1: Table S1. Statistical information to Fig. 3. ANOVA *F* and *P* values, and *post-hoc* Tukey’s honest significance test *P* values of significant increases in IgG against rFh12, rFh15 or rFh15b antigens in vaccinated BALB/c mice using natural and recombinant FABP (nFh12, rFh15 or rFh15b) formulated with the adjuvant adaptation (ADAD) vaccination system with the natural immunomodulator PAL or the synthetic AA0029 compared to untreated control group. (DOCX 17 kb) Additional file 2: Table S2. Statistical information to Fig. 4. ANOVA *F* and *P* values, and *post-hoc* Tukey’s honest significance test *P* values of significant increases in IgG, IgG1, IgG2a against SoSmAWA antigen in vaccinated BALB/c mice using natural and recombinant FABP (nFh12, rFh15 or rFh15b) formulated with the adjuvant adaptation (ADAD) vaccination system with the natural immunomodulator PAL or the synthetic AA0029 compared to untreated control group. (DOCX 19 kb) Additional file 3: Table S3. Complementary statistical information to Fig. 5. Kruskal-Wallis *χ* ^*2*^ degrees of freedom (*df*) and *P-*values, and comparison in pairs *P* values of significant differences in IgG, IgG1, IgG2a, IgE and IgM production against SoSmAWA antigen in vaccinated mice compared to untreated control group at week six of the experiment. (DOCX 16 kb)

## Abbreviations

ADAD: Adjuvant adaptation vaccination system
ANOVA: analysis of variance
FABP: fatty acid binding proteins
nFh12: native FABP of 12 kDa
Qs: non haemolytic saponines from *Quillaja saponaria*
rFh15: recombinant FABP of 15 kDa expressed in *Escherichia coli*
rFh15b: recombinant FABP of 15 kDa expressed in *Trichoplusia ni*
SoSmAWA: soluble adult worm antigens from *Schistosoma mansoni*
