# Supplementary material for: Protection against Schistosoma mansoni infection using a Fasciola hepatica-derived fatty acid binding protein from different delivery systems
Source: Parasit Vectors. 2016 Apr 18;9:216. doi: 10.1186/s13071-016-1500-y (PMC4836169; doi:10.1186/s13071-016-1500-y)
Supplement: Additional file 2: Table S2. — Statistical information to Fig. 4. ANOVA F and P values, and post-hoc Tukey’s honest significance test P values of significant increases in IgG, IgG1, IgG2a against SoSmAWA antigen in vaccinated BALB/c mice using natural and recombinant FABP (nFh12, rFh15 or rFh15b) formulated with the adjuvant adaptation (ADAD) vaccination system with the natural immunomodulator PAL or the synthetic AA0029 compared to untreated control group. (DOCX 19 kb) [file 13071_2016_1500_MOESM2_ESM.docx]

**Table S2. Statistical information to Figure 4**. ANOVA *F* and *P* values, and *post-hoc* Tukey’s honest significance test *P* values of significant increases in IgG, IgG1, IgG2a against SoSmAWA antigen in vaccinated BALB/c mice using natural and recombinant FABP (nFh12, rFh15 or rFh15b) formulated with the adjuvant adaptation (ADAD) vaccination system with the natural immunomodulator PAL or the synthetic AA0029 compared to untreated control group.

| Groups | IgG | IgG1 | IgG2a |
| --- | --- | --- | --- |
| *Experiment 1* |  |  |  |
| Infected | *P* < 0.001 | *P* < 0.001 | ns |
| PAL+Qs | *P* < 0.001 | *P* < 0.001 | ns |
| PAL+Qs+nFh12 | *P* < 0.001 | *P* < 0.001 | *P* = 0.046 |
| ANOVA | *F*_(3,32) =_ 10.99, *P* < 0.001 | *F*_(3,32) =_ 10.20, *P* < 0.001 | *F*_(3,32) =_ 4.50, *P* < 0.001 |
| *Experiment 2* |  |  |  |
| Infected | *P* < 0.001 | *P* = 0.002 | ns |
| PAL+Qs | *P* < 0.001 | *P* = 0.001 | ns |
| PAL+Qs+rFh15 | *P* < 0.001 | *P* < 0.001 | *P* = 0.026 |
| ANOVA | *F*_(3,32) =_ 12.56, *P* < 0.001 | *F*_(3,32) =_ 19.76, *P* < 0.001 | *F*_(3,32) =_ 8.97, *P* < 0.001 |
| *Experiment 3* |  |  |  |
| Infected | *P* < 0.001 | *P* < 0.001 | ns |
| AA0029+Qs | *P* = 0.001 | *P* < 0.001 | ns |
| AA0029+Qs+rFh15 | *P* < 0.001 | *P*= 0.001 | ns |
| AA0029+Qs+rFh15b | *P* < 0.001 | *P* < 0.001 | ns |
| ANOVA | *F*_(4,40) =_ 19.89, *P* < 0.001 | *F*_(4,40) =_ 11.60, *P* < 0.001 | *F*_(4,40) =_ 0.86, ns |

NR, no-reduction: * Significant differences in comparison with infected controls.
