# Supplementary material for: Protection against Schistosoma mansoni infection using a Fasciola hepatica-derived fatty acid binding protein from different delivery systems
Source: Parasit Vectors. 2016 Apr 18;9:216. doi: 10.1186/s13071-016-1500-y (PMC4836169; doi:10.1186/s13071-016-1500-y)
Supplement: Additional file 3: Table S3. — Complementary statistical information to Fig. 5. Kruskal-Wallis χ 2 degrees of freedom (df) and P-values, and comparison in pairs P values of significant differences in IgG, IgG1, IgG2a, IgE and IgM production against SoSmAWA antigen in vaccinated mice compared to untreated control group at week six of the experiment. (DOCX 16 kb) [file 13071_2016_1500_MOESM3_ESM.docx]

**Table S3.** **Complementary statistical information to Figure 5**. Kruskal-Walis χ^2^ degrees of freedom (*df*) and *P* values, and comparison in pairs *P* values of significant differences in IgG, IgG1, IgG2a, IgE and IgM production against SoSmAWA antigen in vaccinated mice compared to untreated control group at week six of the experiment.

| Immunoglobulin | Kruskal-Wallis | | | Untreated  *vs*  AA0029+Qs+rFh15 | Untreated  *vs*  AA0029+Qs+rFh15b | AA0029+Qs+rFh15  *vs*  AA0029+Qs+rFh15b |
| --- | --- | --- | --- | --- | --- | --- |
|  | **χ^2^** | *df* | *P* |  |  |  |
| IgG | 7.96 | 3 | 0.047 | *P* < 0.001 | *P* < 0.001 | ns |
| IgG1 | 16.24 | 3 | 0.001 | *P* < 0.001 | *P* < 0.001 | *P* = 0.018 |
| IgG2a | 10.91 | 3 | 0.012 | *P* = 0.048, | *P* = 0.049 | ns |
| IgE | 12.67 | 3 | 0.005 | *P* = 0.021 | *P* = 0.048 | *ns* |
| IgM | 15.11 | 3 | 0.002 | *P* < 0.001 | *P* < 0.001 | *P* = 0.010 |

Not significant (ns)
